# Field Experiment Effect on Citrus Spider Mite *Panonychus citri* of Venom from Jellyfish *Nemopilema nomurai*: The Potential Use of Jellyfish in Agriculture

**DOI:** 10.3390/toxins13060411

**Published:** 2021-06-10

**Authors:** Huahua Yu, Rongfeng Li, Xueqin Wang, Yang Yue, Song Liu, Ronge Xing, Pengcheng Li

**Affiliations:** 1 CAS and Shandong Province Key Laboratory of Experimental Marine Biology, Center for Ocean Mega-Science, Institute of Oceanology, Chinese Academy of Sciences, No. 7 Nanhai Road, Qingdao 266071, China; rongfengli@qdio.ac.cn (R.L.); xueqinwang@qdio.ac.cn (X.W.); yueyang@qdio.ac.cn (Y.Y.); sliu@qdio.ac.cn (S.L.); xingronge@qdio.ac.cn (R.X.); 2 Laboratory for Marine Drugs and Bioproducts, Pilot National Laboratory for Marine Science and Technology, No. 1 Wenhai Road, Qingdao 266237, China

**Keywords:** cnidarian venom, acaricidal activity, field efficacy

## Abstract

Jellyfish are rich in resources and widely distributed along coastal areas. As a potential approach to respond to jellyfish blooms, the use of jellyfish-derived products is increasing. The citrus spider mite (*Panonychus citri*) is one of the key citrus pests, negatively impacting the quality and quantity of oranges. Due to the resistance and residue of chemical acaricides, it is important to seek natural substitutes that are environmentally friendly. The field efficacy of the venom from the jellyfish *Nemopilema nomurai* against *P. citri* was assayed in a citrus garden. The frozen *N. nomurai* tentacles were sonicated in different buffers to isolate the venom. The venom isolated by PBS buffer (10 mM, pH 6.0) had the strongest acaricidal activity of the four samples, and the corrected field efficacy 7 days after treatment was up to 95.21%. This study demonstrated that jellyfish has potential use in agriculture.

## 1. Introduction

The citrus spider mite (*Panonychus citri*) is one of the key citrus pests. They use a pierce-sucking strategy by piercing the cell walls, extracting nutrients and moisture from the leaf. Their feeding causes necrotic or yellowing spots, which, over time, completely yellows the leaf. Severe mite feeding affects both the quality and quantity of oranges, including flower bud formation, the maturity of the oranges, and even the following year’s production [1]. At present, chemical acaricides, such as spirodiclofen, spiromesifen, diafenthiuron, and bifenazate among others, are commonly used to control the citrus spider mite [2]. However, the citrus spider mite is susceptible to resistance due to its strong adaptability and short generation time [3]. In addition, residual agrochemicals affect food safety and the ecological environment, causing widespread concern [4].

Jellyfish are rich in resources and widely distributed along coastal areas [5]. However, the use of jellyfish resources is limited, and most of the jellyfish is discarded because of its high water content and its stings. As a potential approach to respond to jellyfish blooms, the use of jellyfish-derived products is increasing. In China, the jellyfish *Rhopilema esculentum* and *Nemopilema nomurai* are processed by salt and aluminum, and the processed jellyfish has a shelf life of about two years, which makes jellyfish easy to store and transport [6]. Jellyfish is a popular seafood in China because it is nutritious and a popular food item. According to the *Compendium of Materia Medica*, jellyfish are used to treat gynecology, children’s cold and erysipelas, burns, and so on [7]. The jellyfish peptides obtained by enzymolysis technology have antihypertensive and antioxidant activities [8,9]. A jellyfish venom, as a type of marine toxin, might be a valuable resource in drug design and a tool to study cell physiology [10,11,12]. However, because of the major causes of envenomation, the study of jellyfish venom has been focused on the prevention and treatment of stings, including the identification of sting-related toxins [13,14,15], analysis of toxicity [16,17,18], and first-aid for envenomation [19,20].

**Figure 1:**
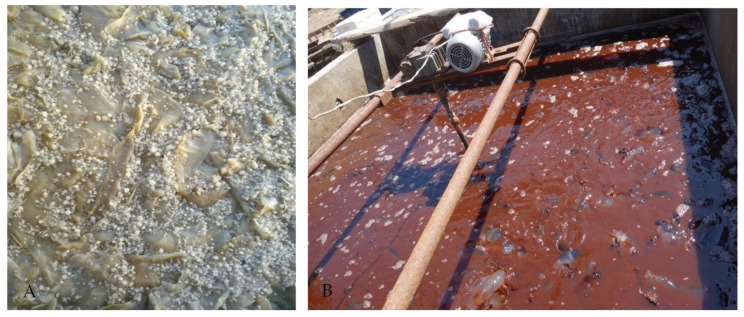
(**A**) Jellyfish processed by salt and alumen; (**B**) jellyfish tentacle autolysis and red liquid containing venom.

Jellyfish *N. nomurai* is the dominant species of jellyfish bloom along coastal areas of China, Japan, and Korea, but only a small amount of this jellyfish is processed as food with salt and aluminum (Figure 1A). A large amount of red liquid containing venom is produced by jellyfish autolysis during the process (Figure 1B). Jellyfish venom has therapeutic potential [11], and venom from *N. nomurai* has anticancer activity [21]. However, this red venom liquid is discarded as a byproduct, which wastes the marine toxin resource. Venoms from other venomous animals, such as spiders, scorpions, cone snails, and wasps, have been reported to have insecticidal activity [22]. As to the use of jellyfish venom in agriculture, we reported the acaricidal activity; the venom from *N. nomurai* had contact toxicity against the spider mite *Tetranychus cinnabarinus* estimated by the FAO-recommended slide-dip method, and the LC_50_ was 29.1 μg/mL [23]. The present study analyzed the field experiment effect of venom from the tentacles of *N. nomurai* to explore the possibility of jellyfish venom as an acaricide. In addition, a new potential use for jellyfish resources may be provided.

## 2. Results and Discussion

The results of the corrected field efficacy are shown in Table 1. It has been reported that the buffers used for isolating venom affect the bioactivities of venom [24,25]. Therefore, the acaricidal activities of four jellyfish venom samples isolated by different buffers were assayed. The four samples (NnFVPBS-1, NnFVPBS-2, NnFVTris-1, and NnFVTris-2) all had acaricidal activity against *P. citri* in the field experiment. NnFVPBS-1 had the strongest acaricidal activity, and the value of the corrected field efficacy 7 days after treatment was up to 95.21%. The values of the corrected field efficacy 1 day, 3 days, 7 days, and 14 days after treatment were 92.51, 91.76, 95.21, and 85.89%, respectively, indicating that NnFVPBS-1 had good persistence. Fourteen days after treatment, the values of the corrected field efficacy for the four samples (NnFVPBS-1, NnFVPBS-2, NnFVTris-1, and NnFVTris-2) were lower than that of 1 day, 3 days, and 7 days after treatment, respectively, and the possible reason is the proliferation of mites or other mites flying in. In addition, the mites’ reduced rate of control 14 days after treatment was −14.63% (due to the possible proliferation of mites or other mites flying in), which made the corrected field efficacy values of all samples 14 days after treatment higher than that of the mites’ reduced rate. The citrus trees treated with samples did not differ greatly in appearance from the citrus trees treated with water, indicating that all samples were harmless to citrus trees 14 days after treatment. The type of buffer affected the acaricidal activity, and the samples NnFVPBS-1 and NnFVPBS-2 isolated by PBS buffer had better control effects than the samples NnFVTris-1 and NnFVTris-2 isolated by Tris buffer. Perhaps the buffers inhibited the activities of both detoxifying and protective enzymes in *P. citri*, and the type and concentration of the buffers affected the acaricidal activity. According to the analysis for significant differences, of the four jellyfish venom samples, only the field efficacy of NnFVPBS-1 had a significant difference vs. PBS-1 (buffer for NnFVPBS-1 isolation). This result showed that the PBS-1 buffer was appropriate for jellyfish venom isolation.

The spider mites are very harmful to plants and the damage affects the yield and quality of food, feed, and fiber. Acaricides are effective ways of controlling spider mites. Most of the acaricides, such as spiromesifen, diafenthiuron, and propargite, are synthetic and remain in the environment. Some natural bioactive substances have been investigated for their acaricidal activity to reduce the residues in the environment. Bamboo tar demonstrated acaricidal activity against *T. cinnabarinus*, and the LC_50_ value was 0.9754 g/L in the greenhouse test, so it might be used as an acaricide in the agricultural field [1]. The fungus isolated from the citrus rust mite, named *Meira geulakonigii* Boekhout, Scorzetti, Gerson & Sztejnberg (Basidiomycota: Ustilaginomycetes), had acaricidal activity against *Phyllocoptruta oleivora*, *P. citri*, and *T. cinnabarinus* [26].

Venom has the potential to be a novel source of bioinsecticides or bioacaricides. Some latrotoxins, pore-forming proteins isolated from spider venom, have the potential as highly-specific insecticides [27]. OAIP-1 from venom of the Australian tarantula *Selenotypus plumipes* had the high insecticidal activity against the agronomically important cotton bollworm *Helicoverpa armigera*, and the oral LD_50_ was 104.2 ± 0.6 pmol/g [28]. Two insecticidal toxins, Ct1a and Ct1b, were isolated from the venom of the Australian theraphosid spider *Coremiocnemis tropix*, and they were lethal to blowflies within 24 h of injection [29]. Four peptides named G1, G3, W3-desK, and W4 were highly active against crickets with an LD_50_ < 130 µg peptide/g animal weight [30]. Six conotoxins with potential insecticidal activity were screened out from a conotoxin library, and the insect bioassay indicated their insecticidal activity against mealworms [31]. Venom from *N. nomurai* had acaricidal activity against *T. cinnabarinus* in the slide-dip method and had insecticidal activity against cotton bollworm *H. armigera* in the diet incorporation assay [23,32]. *Palythoa caribaeorum* venom was lethal to crickets, and the LD_50_ values at 24 h and 48 h were 50.92 ± 10.85 μg protein/g and 3.78 ± 0.243 μg protein/g, respectively [33]. In addition, venom peptides which have the structural scaffolds found in insecticidal toxins, such as inhibitor cysteine knot, defensin-like, and cysteine-stabilized αβ, may have their potential for development as insecticides [22].

In recent years, jellyfish blooms have been a serious global marine ecological disaster, which seriously affect fisheries, ecological environments, tourism, and power plants [34]. Reducing the biomass of jellyfish and using jellyfish resources are the main approaches to deal with jellyfish blooms. Eliminating polyps and intercepting and cutting jellyfish into pieces have been tried to control jellyfish blooms by reducing jellyfish biomass. In Korea, high-pressure water guns have been used successfully to eliminate polyps of the jellyfish *Aurelia aurita*. It is necessary and important to find the polyps, but searching for them is difficult [35]. Jellyfish produce venom for defense, prey capture, and competitor deterrence, and the main components are proteins. A total of 499 proteins with 82 protein groups were identified from *N. nomurai* by jellyfish venomics and venom gland transcriptomics, including phospholipase A_2_, metalloprotease, potassium channel inhibitor, hemolysis, and so on [13]. Although which component in jellyfish venom has acaricidal activity has not been certified, it may be unique to use venom from a “marine pest” to control the terrestrial pests.

Besides venom, collagen is in the mesoglea of jellyfish. Due to good bioavailability and biological properties, jellyfish collagen is a promising candidate in biomedical application [34], such as novel scaffolds and aptasensors for clinical analysis [36,37]. Peptides from the mesoglea of jellyfish have antimicrobial, antioxidant, and hypotensive activity [8,9,38] and also inhibit intracellular tyrosinase and decrease melanin [8], suggesting that peptides from jellyfish may be applied in healthy food and cosmetics. Mucus from jellyfish can accumulate nanoparticles, which may have potential application in the decontamination of nanowater [39].

In summary, venom from *N. nomurai* isolated by PBS buffer had strong acaricidal activity in the field experiment. This result suggested that jellyfish has a potential use in agriculture. However, field experiments are only the beginning of acaricide development, and there is a lot of work to do to develop jellyfish venom as an ideal acaricide. The composition and mechanism for acaricidal activity, insecticidal spectrum and applicability, toxicity, and stability need to be further investigated.

## 3. Materials and Methods

### 3.1. Venom Preparation

*N. nomurai* specimens were collected in Laoshan Bay in Qingdao, Shandong Province, China, in August 2014. The tentacles were manually excised in vivo, packed in polythene bags with ice, and transported to the laboratory. Then, the tentacles were stored at −80 °C until use. The frozen tentacles were sonicated in different cold (4 °C) buffers eight times for 30 s at 100 mV. The fluids were clarified by centrifugation (15,000 g) for 20 min at 4 °C and used as full venom NnFV (NnFVPBS-1, NnFVPBS-2, NnFVTris-1, and NnFVTris-2, where the different suffix letters denote the NnFV sonicated in different buffers listed in Table 2). The concentrations of NnFVPBS-1, NnFVPBS-2, NnFVTris-1, and NnFVTris-2 were determined by the Bradford method [40], using bovine serum albumin (BSA) as a standard.

### 3.2. Field for Experiment

The field for the experiment was located in a citrus garden in Mayang, Hunan Province, China, with an area of approximately 0.4 hectare. Citrus (*Citrus reticulata* Blanco) trees were planted on the mountain. The soil of the mountain was sandy loam with medium fertility and was covered by approximately 1 cm of humus. The citrus trees were well fertilized more than two times per year with 900 kg/ hectare of Yangfeng compound fertilizer, and the citrus trees were 1–1.5 m high. Meteorological conditions on the day of treatment and 7 days after treatment are shown in Table 3.

### 3.3. Efficacy Investigation

To investigate the effect of the buffers used for venom preparation on *P. citri*, four buffers together with four samples were used in the experiment. The concentrations of the four samples (NnFVPBS-1, NnFVPBS-2, NnFVTris-1, and NnFVTris-2) were adjusted to 1.50 mg/mL. The four samples (NnFVPBS-1, NnFVPBS-2, NnFVTris-1, and NnFVTris-2) and buffers (PBS-1, PBS-2, Tris-1, and Tris-2) were diluted 10 times and then sprayed over the entirety of the canopies. Water was used as the control. A manual sprayer with a working pressure of 2–2.3 kg/cm^2^ and a nozzle aperture of 1.2 mm was used. The amount of sample pre-canopies was approximately 0.7 kg. The leaves were entirely moist, but no liquid dripped from the leaves. Three citrus trees were used for each treatment for three replications. The number of mites on three branches from different directions of the same tree was checked before treatment and 1 day, 3 days, 7 days, and 14 days after treatment. The mites reduced rate and corrected field efficacy were calculated according to the following equations:

(1) $$\mathrm{Mites}\;\mathrm{reduced}\;\mathrm{rate}\;\%=\frac{\mathrm{the}\;\mathrm{number}\;\mathrm{before}\;\mathrm{treatment}-\mathrm{the}\;\mathrm{nunber}\;\mathrm{after}\;\mathrm{treatment}}{\mathrm{the}\;\mathrm{number}\;\mathrm{before}\;\mathrm{treatment}}\times 100$$

(2) $$\mathrm{Corrected}\;\mathrm{field}\;\mathrm{efficacy}\;\%=\frac{\%\;\mathrm{mites}\;\mathrm{reduced}\;\mathrm{rate}-\%\;\mathrm{control}\;\mathrm{mites}\;\mathrm{reduced}\;\mathrm{rate}}{100-\%\;\mathrm{control}\;\mathrm{mites}\;\mathrm{reduced}\;\mathrm{rate}}\times 100$$

### 3.4. Statistical Analysis

All data are expressed as the mean ± SD of three parallel measurements. The significance of differences between the means of various experimental groups was analyzed by Duncan’s multiple range test using SPSS 21.0, and *p* < 0.05 was considered statistically significant.

## Figures and Tables

**Table 1:** The corrected field efficacy of venom from jellyfish *N. nomurai* against *P. citri*.

Sample	1 Day after Treatment		3 Days after Treatment		7 Days after Treatment		14 Days after Treatment	
	Mites Reduced Rate (%)	Corrected Field Efficacy (%)	Mites Reduced Rate (%)	Corrected Field Efficacy (%)	Mites Reduced Rate (%)	Corrected Field Efficacy (%)	Mites Reduced Rate (%)	Corrected Field Efficacy (%)
NnFVPBS-1	92.95 ± 3.32 ^a^	92.51 ± 3.32 ^a^	93.51 ± 3.74 ^a^	91.76 ± 4.75 ^a^	95.51 ± 5.08 ^a^	95.21 ± 5.43 ^a^	83.83 ± 2.49 ^a^	85.89 ± 2.17 ^a^
PBS-1	54.82 ± 8.01 ^c^	54.82 ± 8.01 ^c^	61.42 ± 4.57 ^c^	50.98 ± 5.81 ^c^	65.36 ± 3.38 ^a^	62.96 ± 3.62 ^a^	51.19 ± 4.74 ^b^	57.28 ± 4.11 ^b^
NnFVPBS-2	74.16 ± 7.32 ^abc^	74.16 ± 7.32 ^abc^	80.97 ± 9.32 ^ab^	75.82 ± 11.85 ^ab^	65.06 ± 11.71 ^a^	62.64 ± 12.53 ^a^	46.76 ± 7.53 ^b^	53.56 ± 6.57 ^b^
PBS-2	64.51 ± 18.36 ^bc^	64.51 ± 18.35 ^bc^	72.03 ± 9.09 ^bc^	64.46 ± 11.55 ^bc^	60.95 ± 40.00 ^a^	58.24 ± 42.78 ^a^	20.89 ± 15.81 ^b^	30.98 ± 13.79 ^b^
NnFVTris-1	74.45 ± 2.63 ^abc^	74.45 ± 2.63 ^abc^	79.38 ± 9.04 ^ab^	73.79 ± 11.49 ^ab^	70.28 ± 25.14 ^a^	68.22 ± 26.88 ^a^	46.04 ± 7.86 ^b^	52.93 ± 6.07 ^b^
Tris-1	63.99 ± 11.26 ^bc^	63.98 ± 11.26 ^bc^	76.00 ± 10.99 ^bc^	69.51 ± 13.96 ^bc^	64.19 ± 4.98 ^a^	61.71 ± 5.33 ^a^	45.59 ± 9.98 ^b^	52.53 ± 6.86 ^b^
NnFVTris-2	79.64 ± 14.67 ^ab^	79.64 ± 14.67 ^ab^	70.01 ± 10.06 ^bc^	61.89 ± 12.78 ^bc^	69.16 ± 9.56 ^a^	67.03 ± 10.22 ^a^	44.83 ± 47.18 ^b^	51.87 ± 8.71 ^b^
Tris-2	68.55 ± 19.70 ^bc^	68.55 ± 19.70 ^bc^	61.50 ± 7.48 ^c^	51.07 ± 9.50 ^c^	63.73 ± 5.88 ^a^	61.22 ± 6.28 ^a^	36.26 ± 29.77 ^b^	44.39 ± 41.16 ^b^

Note: Different letters indicate significant differences at *p* < 0.05. The values of mites reduced rate (%) of control (water) were 0, 21.30, 6.48, and −14.63 % after 1 day, 3 days, 7 days, and 14 days treatment.

**Table 2:** Samples of venom from jellyfish *N. nomurai* obtained by different buffers.

Samples	Buffer
NnFVPBS-1	PBS-1 (10 mM, pH 6)
NnFVPBS-2	PBS-2 (10 mM, pH 6, 1 mM GSH + 5mM NaCl)
NnFVTris-1	Tris-1 (50 mM, pH 7.8)
NnFVTris-2	Tris-2 (50 mM, pH 7.8, 1mM GSH + 5mM NaCl)

**Table 3:** Meteorological conditions on the day of treatment and 7 days after treatment.

Date (M/D)	Average Temperature (℃)	Relative Humidity (%)	Amount of Precipitatiom (mm)
6/18	28.6	72	-
6/19	29.2	96	6
6/20	29.4	74	-
6/21	28.9	100	10
6/22	30.8	72	-
6/23	31.4	69	-
6/24	30.4	56	-
6/25	29.4	69	-

## Data Availability

The data presented in this study are available on request from the corresponding author. The data are not publicly available due to privacy.
